# Tonabersat Significantly Reduces Disease Progression in an Experimental Mouse Model of Multiple Sclerosis

**DOI:** 10.3390/ijms242417454

**Published:** 2023-12-14

**Authors:** Andrea Kwakowsky, Bhavya Chawdhary, Antonio de Souza, Emily Meyer, Andrew H. Kaye, Colin R. Green, Stanley S. Stylli, Helen Danesh-Meyer

**Affiliations:** 1 Department of Anatomy and Medical Imaging, Centre for Brain Research, Faculty of Medical and Health Science, University of Auckland, Auckland 1023, New Zealand; b.chawdhary@auckland.ac.nz (B.C.); antonio.desouza@auckland.ac.nz (A.d.S.); edan547@aucklanduni.ac.nz (E.M.); 2 Pharmacology and Therapeutics, Galway Neuroscience Centre, School of Medicine, Ollscoil na Gaillimhe—University of Galway, H91 W5P7 Galway, Ireland; 3 Department of Opthalmology, Faculty of Medical and Health Sciences, University of Auckland, Auckland 1023, New Zealand; c.green@auckland.ac.nz (C.R.G.); h.daneshmeyer@auckland.ac.nz (H.D.-M.); 4 Department of Surgery, University of Melbourne, Melbourne, VIC 3010, Australia; kaye.andrew@gmail.com (A.H.K.); stanley.stylli@mh.org.au (S.S.S.); 5 Department of Neurosurgery, Hadassah Hebrew University Hospital, Jerusalem 91120, Israel; 6 Department of Neurosurgery, The Royal Melbourne Hospital, Parkville, VIC 3052, Australia

**Keywords:** multiple sclerosis, Connexin 43, tonabersat, inflammasome, brain

## Abstract

Multiple sclerosis (MS) is a neurodegenerative disease marked by chronic neuroinflammation thought to be mediated by the inflammasome pathway. Connexin 43 (Cx43) hemichannels contribute to the activation of the inflammasome through the release of adenosine triphosphate (ATP) inflammasome activation signals. The objective of the study was to evaluate if the Cx43 hemichannel blocker, tonabersat, is effective in modulating the inflammatory response and reducing disability in the myelin oligodendrocyte glycoprotein 35–55-induced experimental autoimmune encephalomyelitis (MOG_35–55_ EAE) model of MS. Here, we show that the Cx43 hemichannel blocking drug, tonabersat, significantly reduced expression of neuroinflammatory markers for microglial activation (ionized calcium-binding adapter molecule 1 (Iba1)) and astrogliosis (glial fibrillary acidic protein (GFAP)) while preserving myelin basic protein (MBP) expression levels in the corpus callosum, motor cortex, and striatum regions of the brain in MOG_35–55_ EAE mice. Reduced NOD-like receptor protein 3 (NLRP3) inflammasome complex assembly and Caspase-1 activation confirmed the drug’s mode of action. MOG_35–55_ EAE mice showed clinical signs of MS, but MOG_35–55_ EAE mice treated with tonabersat retained behavior closer to normal. These data suggest that clinical trial phase IIb-ready tonabersat may merit further investigation as a promising candidate for MS treatment.

## 1. Introduction

Multiple sclerosis (MS) is a chronic inflammatory demyelinating neurodegenerative disease of the central nervous system (CNS). It is the most frequent cause of neurological disability in young adults [1]. Current therapeutics used to treat disease onset, progression, and symptoms often have varying efficacy and adverse effects, impeding their administration and prolonged use. Novel therapeutics are required to help provide a safe, enduring therapeutic effect.

Neuroinflammation has been identified to be a major factor in the neuropathology of MS, with inflammasome activation playing a critical role in the neuroinflammatory process [2,3,4,5,6]. Inflammasomes are protein complexes that can be activated by a wide range of stimuli [7]. The NOD-like receptor protein 3 (NLRP3) inflammasome is the most widely studied of the inflammasomes and has been shown to play a role in the neuropathology of MS [3,4,5,6,8,9,10,11,12,13]. Furthermore, in patients with MS, it has been shown that memory T cells exhibit a reduced ability to suppress NLRP3 inflammasome activation [14]. Inflammasome activation and subsequent release of inflammatory cytokines is a two-step process involving a priming signal and an activation signal [15]. Multiple priming signals have been proposed with a small number of potential activation signals [15]. Adenosine triphosphate (ATP) released through pathologically open Connexin 43 (Cx43) hemichannels is one key activation signal [16].

We hypothesized that modulating the inflammasome pathway by blocking Cx43 hemichannels to prevent the release of the activating signal ATP from cells has the potential to offer a new treatment paradigm for MS [17]. In several CNS disease models, such as spinal cord injury, retinal injury, brain ischemia, Amyotrophic Lateral Sclerosis, Parkinson’s, and Alzheimer’s disease [18,19,20], blocking Cx43 hemichannel opening significantly reduced the disease signs and pathology [16,17,18]. We have previously shown that under pathological conditions, Cx43 hemichannels located in the cell membrane open prematurely, forming a ‘pathological pore’, allowing the release of ATP from the cell [21,22]. ATP moving to the extracellular space can act as an inflammasome activator signal [16]. One connexin hemichannel blocker is tonabersat (also known as xiflam), a novel benzopyran compound (cis-6-acetyl-4S-(3-chloro-4-fluoro-benzoylamino)-3,4-dihydro-2,2-di-methyl-2H-benzo[b]pyrane-3S-ol (SB-220453)). We have previously shown tonabersat to be a Cx43 hemichannel blocker [19,23]. Because the Cx43 hemichannel blockade would act upstream of current treatment modalities for MS, it may enable treatment at an earlier stage of the disease without the adverse side effects associated with many of the current treatment modalities.

Myelin oligodendrocyte glycoprotein 35–55-induced experimental autoimmune encephalomyelitis MOG_35–55_ EAE is an MS mouse model in which the NLRP3 inflammasome plays a pivotal role in the pathogenesis of neuroinflammation [24,25]. Microglia and astrocytes are critical in maintaining the optimal milieu for neuronal function and current literature suggests a crucial role of these glial cells in neuroinflammatory processes and progression of MS [26,27]. The role and activation of these cells are well characterized in the perpetuation of chronic inflammation but also their beneficial actions in health and disease [28,29]. Microglia and astrocyte reactivity are prevalent features of the neuroinflammatory processes that begin during the early stages of the EAE and MS and is characterized by molecular and morphological changes [26,30,31,32,33].

This study was undertaken to evaluate if the Cx43 hemichannel blocker, tonabersat, is effective in modulating the inflammatory response and reducing disability in this MOG_35–55_ EAE mouse model of MS. Immunohistochemical analyses indicated that tonabersat treatment reduced NLRP3 inflammasome activation, Caspase-1 activation, and inflammation in the mouse corpus callosum, motor cortex and striatum. This led to less demyelination and significantly decreased limb paralysis. These findings indicate that tonabersat is a promising candidate to be translated into clinical trials for the treatment of MS.

## 2. Results

### 2.1. Behavioural Changes following MOG_35–55_ EAE and Tonabersat Early Dosing Administration

To examine the effect of tonabersat treatment in MOG_35–55_ EAE mice, daily behavioral scoring was performed on all treatment groups with early and late dosing regimens.

**Figure 1:**
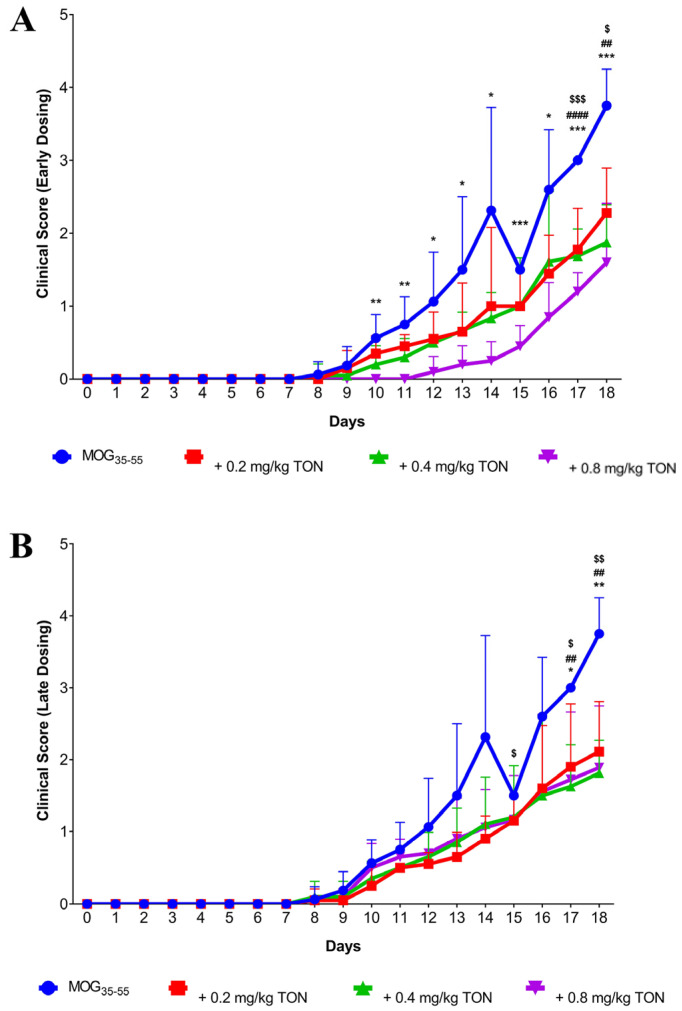
(**A**) Clinical behavioral scoring data of MOG_35–55_ EAE mice, MOG_35–55_ EAE mice with 0.2 mg/kg tonabersat treatment, MOG_35–55_ EAE mice with 0.4 mg/kg tonabersat treatment, and MOG_35–55_ EAE mice with 0.8 mg/kg tonabersat treatment following an early drug dosing regimen. MOG_35–55_ EAE (MOG_35–55_) mice showed the highest mean clinical behavioral score by day 18 compared to the three tonabersat early dosing groups, with MOG_35–55_ EAE mice with 0.8 mg/kg tonabersat treatment (0.8 mg/kg TON) presenting the lowest mean clinical behavioral score. Each data point represents a mean clinical score for each experimental group plotted against time showing standard error representative of each day (Multiple-measure two-way ANOVA; MOG_35–55_ mice *n* = 8, 0.2 mg/kg TON mice (^$^ *p* ≤ 0.05; ^$$$^ *p* ≤ 0.001) *n* = 10, 0.4 mg/kg TON mice (^##^ *p* ≤ 0.01; ^####^ *p* ≤ 0.0001) *n* = 10 and 0.8 mg/kg TON mice (* *p* ≤ 0.05, ** *p* ≤ 0.01, *** *p* ≤ 0.001) *n* = 10)). (**B**) Clinical behavioral scoring data of MOG_35–55_ EAE mice, MOG_35–55_ EAE mice with 0.2 mg/kg tonabersat treatment, MOG_35–55_ EAE mice with 0.4 mg/kg tonabersat treatment, and MOG_35–55_ EAE mice with 0.8 mg/kg tonabersat treatment following a late drug dosing regimen. MOG_35–55_ EAE (MOG_35–55_) mice showed the highest mean clinical behavioral score by day 18 compared to the three tonabersat late dosing groups, with MOG_35–55_ EAE mice with 0.4 mg/kg tonabersat treatment (0.4 mg/kg TON) presenting the lowest mean clinical behavioral score. Each data point represents a mean clinical score for each experimental group plotted against time showing standard error representative of each day (Multiple-measure two-way ANOVA; MOG_35–55_ mice *n* = 8, 0.2 mg/kg TON mice (^$^ *p* ≤ 0.05, ^$$^ *p* ≤ 0.01) *n* = 10, 0.4 mg/kg TON mice (^##^ *p* ≤ 0.01) *n* = 10 and 0.8 mg/kg TON mice (* *p* ≤ 0.05; ** *p* ≤ 0.01) *n* = 10)).

At day 10, MOG_35–55_ EAE mice presented behavior significantly different from MOG_35–55_ EAE mice with early dosing 0.8 mg/kg tonabersat treatment (*p* = 0.0068), with the MOG_35–55_ EAE mice showing clinical signs of MS and the 0.8 mg/kg tonabersat-treated mice retaining normal behavior despite having been MOG_35–55_ challenged (Figure 1A). 

By day 18, the mean clinical behavioral score of MOG_35–55_ EAE mice was significantly higher than the mean clinical behavioral score of MOG_35–55_ EAE mice with 0.8 mg/kg tonabersat treatment (*p* = 0.0009). Based on the clinical EAE scoring guidelines, the mean clinical behavioral score of the MOG_35–55_ EAE group indicated that the mice showed paralysis of the hind limbs, as was made apparent by the dragging of one limb upon ambulation, whereas the mean clinical behavioral score of the MOG_35–55_ EAE mice with 0.8 mg/kg tonabersat treatment indicated the absence of paralysis, confirmed when the mice could be rolled with marginal struggle. The 0.0 mean clinical score obtained by healthy control animals indicated the absence of EAE (Figure 1A).

Further significant differences in clinical behavioral scoring were seen between the MOG_35–55_ EAE group and the MOG_35–55_ EAE mice with 0.4 mg/kg tonabersat treatment at day 17 (*p* < 0.0001) and at day 18 (*p* < 0.0032). Significant differences between the MOG_35–55_ EAE group and MOG_35–55_ EAE mice with 0.2 mg/kg tonabersat treatment were observed at day 17 (*p* = 0.0009) and on day 18 (*p* = 0.0103). At day 18, the 0.4 mg/kg early dosing group shared similar clinical behavior with the 0.8 mg/kg early dosing group (1.8 ± 0.14 and 1.6 ± 0.10). The 0.2 mg/kg tonabersat-treated mice presented with a higher mean clinical behavioral score of 2.27 ± 0.15 at day 18, indicating that the mice displayed no hind limb paralysis upon ambulation but failed to remain upright upon the experimenter trying to roll the animal (Figure 1A).

### 2.2. Behavioural Changes following MOG_35–55_ EAE Onset and Tonabersat Late Dosing Administration

At day 8, all mice started to show some clinical signs of MS, but differences between control and treated groups became increasingly apparent over time, with MOG_35–55_ EAE mice being the most affected. At day 17, the MOG_35–55_ EAE mice exhibited behavior significantly different from MOG_35–55_ EAE mice with late dosing of 0.8 mg/kg tonabersat treatment (*p* = 0.0149) (Figure 1B). Behavioral differences between these two groups further intensified during the following days, whereby at day 18, the mean clinical behavioural score of MOG_35–55_ EAE mice was significantly higher than the mean clinical behavioural score of the MOG_35–55_ EAE mice with 0.8 mg/kg tonabersat treatment (*p* = 0.0031).

The 0.4 mg/kg tonabersat dosing group presented the lowest day 18 clinical behavioral score out of the three-drug dosing groups relative to the clinical behavioral score of the MOG_35–55_ EAE group (1.81 ± 0.14 and 3.75 ± 0.27, *p* = 0.0032) and the low MOG_35–55_ EAE mice with 0.2 mg/kg tonabersat treatment was more affected than the other two treatment groups. A significant difference in clinical behavioral scores between the 0.4 mg/kg tonabersat dosing group and the MOG_35–55_ EAE untreated group was also found on day 17 (*p* = 0.0149). Significant differences between the MOG_35–55_ EAE group and MOG_35–55_ EAE mice with 0.2 mg/kg tonabersat treatment were observed at days 15 (*p* = 0.0394), 17 (*p* = 0.0141) and 18 (*p* = 0.0056) (Figure 1B).

At day 18, the 0.4 mg/kg late tonabersat dosing group shared similar clinical behavioral scores with the 0.8 mg/kg tonabersat late dosing group (1.8 ± 0.14 and 1.81 ± 0.15). The 0.2 mg/kg tonabersat-treated mice presented a mean clinical behavioral score of 2.11 ± 0.16 at day 18, indicating that the mice displayed no hind limb paralysis upon ambulation but failed to remain upright upon the experimenter trying to roll the animal (Figure 1B).

Both early and late dosing treatments were used to identify if tonabersat produced different effects in behavior when administered at two different starting time points. No significant differences between the mean clinical behavioral scores of each respective early and late dosing group were observed. Early dosing clinical scores of 0.2 mg/kg, 0.4 mg/kg and 0.8 mg/kg mice at the end point of day 18 were not significantly different from the late dosing clinical scores of 0.2 mg/kg, 0.4 mg/kg and 0.8 mg/kg mice, respectively. However, as the early dosing 0.8 mg/kg tonabersat group showed slightly better improvements in clinical scores (Supplementary Table S4), all immunohistochemistry experiments were performed on tissue from this treatment group.

### 2.3. Immunohistochemical Analysis of MBP, NLRP3, Caspase-1, Iba-1 and GFAP in the Corpus Callosum of Control, MOG_35–55_ EAE, and MOG_35–55_ EAE Mice with 0.8 mg/kg Tonabersat Treatment

**Figure 2:**
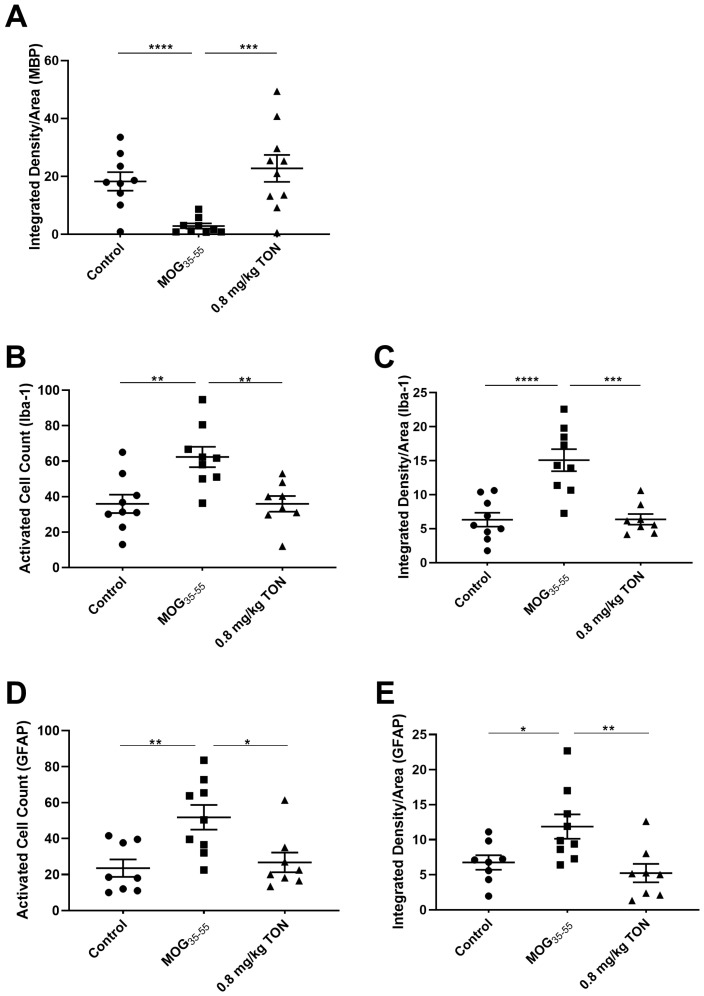
Mean integrated density of MBP and mean activated cell count and mean integrated density of Iba-1 and GFAP in the corpus callosum of control, MOG_35–55_ EAE mice, and MOG_35–55_ EAE mice with 0.8 mg/kg tonabersat treatment. The corpus callosum of control mice, MOG_35–55_ EAE (MOG_35–55_) mice, and MOG_35–55_ EAE mice with 0.8 mg/kg tonabersat treatment (0.8 mg/kg TON) were immunostained with MBP, Iba-1, and GFAP. MBP (**A**) integrated densities of MOG_35–55_ mice showed a significant decrease compared to both control and 0.8 mg/kg TON mice. Control mice and 0.8 mg/kg TON mice shared similar means with no significant differences between the two groups for MBP expression. Activated microglia and astrocytic cell counts (**B**,**D**), and Iba-1 and GFAP integrated densities (**C**,**E**) of MOG_35–55_ mice showed a significant increase compared to both control and 0.8 mg/kg TON mice. There were no significant differences between the control mice and 0.8 mg/kg TON mice for either inflammatory marker. Data points expressed as mean values ± SEM (one-way ANOVA and Tukey’s post-hoc test (* *p* ≤ 0.05, ** *p* ≤ 0.01, ***p ≤ 0.001, ****p ≤ 0.0001; control mice *n* = 8–9, MOG_35–55_-treated mice *n* = 9 and 0.8 mg/kg TON mice *n* = 8–10)).

**Figure 3:**
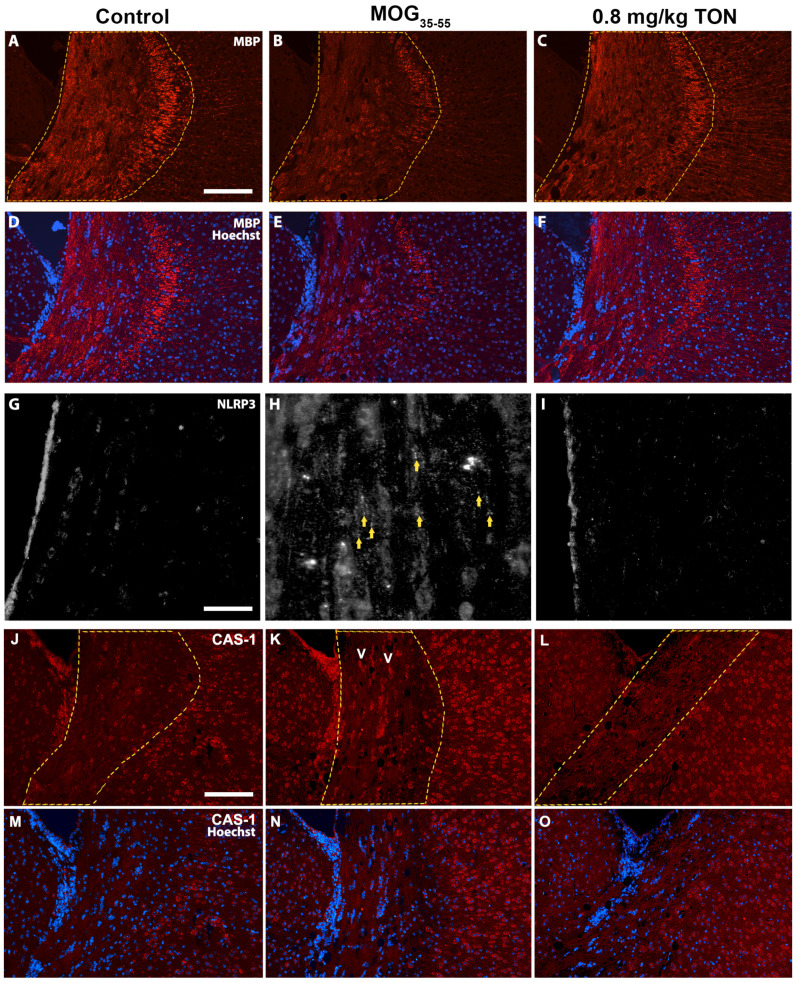
(**A**–**F**) Photomicrographs showing the effects of tonabersat on MBP expression within the mouse corpus callosum. Representative images depicting the immunolabeling of MBP (red) and Hoechst (blue) within the corpus callosum region (outlined in yellow dotted line) of the mouse brain in control (**A**,**D**), MOG_35–55_ EAE (MOG_35–55_) mice (**B**,**E**), and MOG_35–55_ EAE mice with 0.8 mg/kg tonabersat treatment (0.8 mg/kg TON) (**C**,**F**). MOG_35–55_-treated mice showed a significant reduction in MBP expression, while the 0.8 mg/kg TON-treated mice showed MBP expression levels parallel to control mice. Scale bar = 200 μm. (**G**–**I**) Representative photomicrographs of NLRP3 inflammasome labeling in the mouse corpus callosum. Images showing the NLRP3 immunolabeling within the corpus callosum region of the mouse brain in control (**G**), MOG_35–55_ mice (**H**), and 0.8 mg/kg TON mice (**I**). MOG_35–55_ mice displayed the greatest level of NLRP3 labeling (marked with yellow arrows). Scale bar = 100 μm. (**J**–**O**) Representative photomicrographs of Caspase-1 labeling in the mouse corpus callosum. Images showing the Caspase-1 (CAS-1) immunolabeling within the corpus callosum region of the mouse brain in control (**J**,**M**), MOG_35–55_ mice (**K**,**N**), and 0.8 mg/kg TON mice (**L**,**O**). MOG_35–55_ mice displayed the greatest level of Caspase-1 labeling (see arrowheads). Scale bar = 200 μm.

Loss of MBP is one of the main characteristics of MS progression in patients and EAE animal models [34,35,36,37,38,39]. The mean integrated density of MBP was significantly decreased throughout the brain, with the most significant changes observed in the corpus callosum of MOG_35–55_ EAE mice in contrast to control mice (*p* = 0.0123), but significantly protected in MOG_35–55_ EAE mice with 0.8 mg/kg tonabersat treatment compared to the MOG_35–55_ EAE mice (*p* = 0.0010). No significant differences in the MBP integrated density means were observed between the control and 0.8 mg/kg tonabersat groups (Figure 2A and Figure 3A–F).

Immunohistochemistry against NLRP3 in the mouse corpus callosum revealed that MOG_35–55_ EAE mice have a greater level of NLRP3 inflammasome complex assembly and Caspase-1 labeling compared to control mice. A difference in NLRP3 and Caspase-1 immunolabeling is also seen between MOG_35–55_ EAE mice and MOG_35–55_ EAE mice with 0.8 mg/kg tonabersat treatment, with 0.8 mg/kg tonabersat mice treatment resulting in a major reduction in NLRP3 inflammasome activation compared to MOG_35–55_ EAE mice (Figure 3G–I) and reduced Caspase-1 immunolabeling (Figure 3J–O).

**Figure 4:**
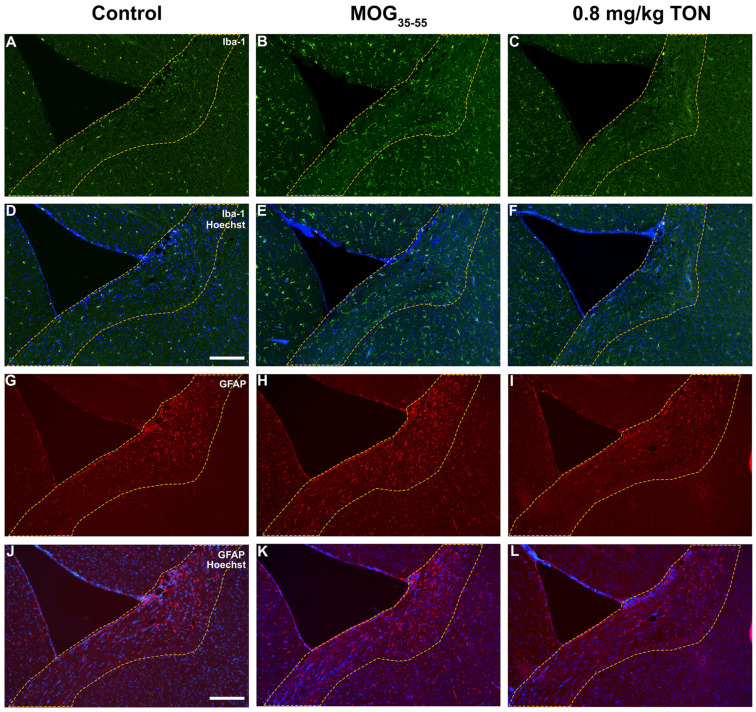
(**A**–**F**) Photomicrographs showing the effects of tonabersat on Iba-1 expression within the mouse corpus callosum. Representative images depicting the immunolabeling of Iba-1 (green) and Hoechst (blue) within the corpus callosum region (outlined in yellow dotted line) of the mouse brain in control (**A**,**D**), MOG_35–55_ EAE (MOG_35–55_) mice (**B**,**E**), and MOG_35–55_ EAE mice with 0.8 mg/kg tonabersat treatment (0.8 mg/kg TON) (**C**,**F**). MOG_35–55_ mice showed the greatest level of Iba-1 expression, while the expression was significantly decreased in the 0.8 mg/kg TON mice and comparable to that of the control mice. Scale bar = 200 μm. (**G**–**L**) Photomicrographs showing the effects of tonabersat on GFAP expression within the mouse corpus callosum. Representative images depicting the immunolabeling of GFAP (red) and Hoechst (blue) within the corpus callosum region (outlined in yellow dotted line) of the mouse brain in control (**G**,**J**), MOG_35–55_ mice (**H**,**K**), and 0.8 mg/kg TON (**I**,**L**). MOG_35–55_-treated mice showed the greatest level of GFAP expression, while the 0.8 mg/kg TON mice showed a significant reduction in inflammation with GFAP expression similar to control mice. Scale bar = 200 μm.

Quantification of activated ionized calcium-binding adapter molecule 1 (Iba-1) positive microglia in the corpus callosum showed that MOG_35–55_ EAE mice exhibited a significant increase in Iba-1 activated cell count in comparison to control mice (*p* = 0.0037) and that the Iba-1 activated cell count was significantly reduced in MOG_35–55_ EAE mice with 0.8 mg/kg tonabersat treatment compared to the MOG_35–55_ mice (*p* = 0.0048). In the corpus callosum, MOG_35–55_ EAE mice exhibited significantly increased Iba-1 mean integrated density compared to control mice (*p* < 0.0001), and the MOG_35–55_ EAE 0.8 mg/kg tonabersat-treated group had significantly decreased mean integrated density compared to the MOG_35–55_ administered mice (*p* = 0.0001). No significant difference in Iba-1 activated cell counts and integrated density means was found between the control and the MOG_35–55_ EAE mice with 0.8 mg/kg tonabersat treatment (Figure 2B,C and Figure 4A–F).

Quantification of activated glial fibrillary acidic protein (GFAP)-positive astrocytes in the corpus callosum revealed that MOG_35–55_ EAE mice exhibited a significant increase in GFAP-activated cell count in comparison to control mice (*p* = 0.0067) and that the GFAP-activated cell count was significantly reduced in MOG_35–55_ EAE mice with 0.8 mg/kg tonabersat treatment compared to the MOG_35–55_ EAE mice (*p* = 0.0165). In the corpus callosum, the MOG_35–55_ EAE mice exhibited significantly increased GFAP integrated density compared to the control (*p* < 0.0454), and the MOG_35–55_ EAE mice with 0.8 mg/kg tonabersat treatment showed significantly decreased integrated density compared to the MOG_35–55_ EAE mice (*p* = 0.0086). No significant difference in GFAP-activated cell counts and integrated density was found between the control and 0.8 mg/kg tonabersat-treated groups (Figure 2D,E and Figure 4G–L).

### 2.4. Immunohistochemical Analysis of Iba-1 and GFAP in the Motor Cortex of Control, MOG_35–55_ EAE and MOG_35–55_ EAE Mice with 0.8 mg/kg Tonabersat Treatment

Quantification of activated Iba-1 microglia in the motor cortex revealed that t MOG_35–55_ EAE mice exhibited a significant increase in Iba-1 activated cell count in comparison to control mice (*p* = 0.0174) and that the Iba-1 activated cell count was significantly reduced in MOG_35–55_ EAE mice with 0.8 mg/kg tonabersat treatment compared to the MOG_35–55_ EAE mice (*p* = 0.0070). Iba-1 immunolabeling revealed that the 0.8 mg/kg tonabersat-treated group exhibited significantly reduced integrated density compared to the MOG_35–55_ EAE mice (*p* = 0.0350). No significant differences were noted between the control and the MOG_35–55_ EAE mice with 0.8 mg/kg tonabersat treatment for Iba-1 activated cell counts or integrated density. There was no difference between MOG_35–55_ EAE and control groups for Iba-1 integrated density (Supplementary Figures S1A,B and S2A–F).

Quantification of activated GFAP astrocytes in the motor cortex revealed that MOG_35–55_ EAE mice exhibited a significant increase in GFAP-activated cell count in comparison to control mice (*p* = 0.0005) and that the GFAP-activated cell count was significantly reduced in MOG_35–55_ EAE mice with 0.8 mg/kg tonabersat treatment compared to the MOG_35–55_ EAE mice (*p* < 0.5627). In the corpus callosum, MOG_35–55_ EAE mice exhibited significantly increased GFAP integrated density compared to control mice (*p* = 0.0050), and the MOG_35–55_ EAE mice with 0.8 mg/kg tonabersat treatment showed significantly lower integrated density compared to the MOG_35–55_ EAE mice (*p* = 0.0056). No significant difference in GFAP-activated cell counts and integrated density was found between the control and the MOG_35–55_ EAE mice with 0.8 mg/kg tonabersat treatment (Supplementary Figures S1C,D and S2G–L).

### 2.5. Immunohistochemical Analysis of Iba-1 and GFAP in the Striatum of Control, MOG_35–55_ EAE and MOG_35–55_ EAE Mice with 0.8 mg/kg Tonabersat Treatment

Quantification of activated Iba-1 positive microglia in the striatum revealed that MOG_35–55_ EAE mice exhibited a significant increase in Iba-1 activated cell count in comparison to control mice (*p* = 0.0224), and that the Iba-1 activated cell count was significantly lower in MOG_35–55_ EAE mice with 0.8 mg/kg tonabersat treatment compared to the MOG_35–55_ EAE mice (*p* = 0.3713). In the corpus callosum, MOG_35–55_ EAE mice exhibited significantly increased Iba-1 integrated density compared to the control mice (*p* < 0.0001), and the 0.8 mg/kg tonabersat-treated group showed significantly decreased integrated density compared to the MOG_35–55_ EAE untreated mice (*p* < 0.0001). No significant difference in Iba-1 activated cell counts and integrated density was found between the control and 0.8 mg/kg tonabersat treatment groups (Supplementary Figures S3A,B and S4A–F).

Quantification of activated GFAP positive astrocyte in the striatum revealed that MOG_35–55_ EAE mice exhibited a significant increase in GFAP-activated cell count in comparison to control mice (*p* = 0.0101) and that the GFAP-activated cell count was significantly lower in MOG_35–55_ EAE mice with 0.8 mg/kg tonabersat treatment compared to the MOG_35–55_ EAE mice (*p* = 0.0188). In the corpus callosum, MOG_35–55_ EAE mice exhibited significantly increased GFAP integrated density compared to the control mice (0.12 ± 0.02 versus 0.05 ± 0.01, *p* = 0.0109), and the 0.8 mg/kg tonabersat-treated group showed significantly decreased integrated density compared to the untreated MOG_35–55_ EAE mice (*p* = 0.0206). No significant difference in GFAP-activated cell counts and integrated density was found between the control and 0.8 mg/kg tonabersat groups (Supplementary Figures S3C,D and S4G–L).

## 3. Discussion

MS is a neurodegenerative disease marked by chronic neuroinflammation thought to be mediated by the inflammasome pathway. Inflammasome activation involves a priming signal and an activation signal [15]. Whilst multiple priming signals have been proposed, there are only a few potential activation signals [15]. ATP released through pathologically open Cx43 hemichannels is one key activation signal [16]. In this study, we, therefore, set out to test the hypothesis that the Cx43 hemichannel blocker, tonabersat, will ameliorate signs of MS in the MOG_35–55_ EAE mouse model by preventing inflammasome activation.

In this study, we observed that MOG_35–55_ EAE mice presented with a high level of inflammation and demyelination and had poor clinical behavior outcomes. MOG_35–55_ EAE mice treated with tonabersat, however, showed a reduced inflammatory profile and the demyelination observed in MOG_35–55_ EAE mice was prevented in the tonabersat-treated animals. Tonabersat-treated mice had diminished NLRP3 inflammasome assembly relative to EAE mice, indicating that tonabersat has the effect of reducing NLRP3 inflammasome activation, which would, untreated, drive downstream inflammatory processes. Cx43 hemichannel blockade using tonabersat in this mouse model of MS resulted in a clinical behavioral outcome significantly closer to that of normal control mice.

The timeline of MOG_35–55_ EAE onset and disease progression was strongly in line with previous work carried out by Hasselmann [25], demonstrating that C57BL/6 MOG_35–55_-treated mice show clinical behavioral signs of MS at 10 days post-MOG_35–55_ EAE induction. By the dosing endpoint, 18 days post-EAE induction, strong statistically significant differences were apparent in mouse clinical behavioral scores between control and MOG_35–55_ EAE mice. The magnitude of difference between scores of control and MOG_35–55_ EAE mice confirms that this model recapitulates aspects of MS and provides a useful system to test tonabersat as a potential MS therapeutic. We found a dose-dependent relationship between clinical behavioral scores and tonabersat dosage, with higher doses of tonabersat corresponding with lower clinical behavioral scores compared with untreated MOG_35–55_ EAE mice. No significant differences in clinical scores at 18 days were seen between each respective tonabersat dosing group among early and late dosing groups. These findings may mean that it will be possible to treat patients already displaying signs of MS upon diagnosis yet still obtain therapeutic effects if treatment had begun during the earlier stages of the disease.

Recent studies have reported that tonabersat produces its NLRP3 inflammasome activation reducing effect through Cx43 hemichannel blockade [19,23,40]. Cx43 hemichannel block inhibits the release of intracellular ATP into the extracellular space, preventing NLRP3 inflammasome activation and, as a result, secretion of the pro-inflammatory cytokines interleukin-1 beta (IL-1β) and interleukin-18 (IL-18) [16,19,41]. Specifically, tonabersat has been shown to reduce NLRP3 complex formation in in vitro models, which was complimented with lower levels of Caspase-1 cleavage, confirming the link between ATP release and NLRP3 inflammasome activation [16,19]. In models of dry age-related macular degeneration and diabetic retinopathy, orally delivered tonabersat inhibited loss of retinal function and inflammation in a similar manner to another connexin43 hemichannel blocker, Peptide5 [42,43]. In human-induced pluripotent stem cell-derived astrocytes from both familial and sporadic amyotrophic lateral sclerosis (ALS), both the known hemichannel blocker Gap19 and tonabersat provided neuroprotection and reduced ALS astrocyte-mediated neuronal hyperexcitability [40]. That study extended to chronic administration of tonabersat in a superoxide dismutase type 1^G93A^ (SOD1^G93A^) mouse model, which showed reduced reactive astrocytosis and microgliosis and resulted in significant motor neuron protection [40]. The results in the present study are consistent with those.

A previous study in EAE-induced mice also reported abnormal activation and activity of NLRP3, as well as other markers of inflammasome activation, such as apoptosis-associated speck-like protein containing a caspase recruitment domain (ASC) and pro-caspase 1 [41]. Studies which have used knockout animals have validated the role of the NLRP3 inflammasome in driving the progression of EAE; deficiency of inflammasome complex proteins delayed and reduced the disease progression in EAE and improved clinical scores [44,45]. An NLRP3 gene knockout study using a cuprizone model of MS found that *Nlrp3^−/−^* mice showed a reduction in both astrogliosis and microgliosis [46]. RRx-001, an inhibitor of the NLRP3 inflammasome, has also been shown to attenuate the symptoms of EAE and inflammation [47]. A reduction in activated microglia numbers leads to a drop in immune cell infiltration, such as CD4+ T cells, B cells, macrophages and neutrophils, slowing the progression of EAE [32]. Further studies have revealed that *Nlrp3^−/−^* mice lacking caspase-1 also show delayed demyelination and reduced gliosis [33]. This link between NLRP3 inflammasome activation and clinical disease outcome was further demonstrated in a study of EAE-induced mice showing a delay in disease progression, reduced levels of MOG-specific T cells within the lymph nodes and an improved disease clinical score following a knockout of the ASC protein [48]. Our data in the present study using tonabersat to block hemichannels echo those findings.

Neuroinflammation is a major neuropathological hallmark of MS. The corpus callosum of MOG_35–55_ EAE-treated mice appeared to have the highest immunolabeling density of Iba-1 and GFAP and the highest number of activated microglia and astrocytes, followed by the motor cortex and striatum. These findings are consistent with Hasselmann [25], who showed that the corpus callosum from MOG_35–55_ EAE mice, at the peak of behavioral changes, displayed higher amounts of microglia/leukocyte labeling (assessed with CD45 labeling), increased inflammatory lesions, and exhibited decreased amounts of MBP compared to controls and that these pathological changes were more severe in this region compared to cerebral and cortical areas. The corpus callosum and other white matter regions display a greater dispersal of activated microglia in MS compared to grey matter regions [49]. We found that MOG_35–55_ EAE-treated mice, in all three brain regions of interest, showed significantly greater microglia cell activation and integrated densities compared to MOG_35–55_ EAE mice with 0.8 mg/kg tonabersat treatment whose Iba-1 labeling was similar to the control mice. Several studies have demonstrated the role of microglia activation in the progression of EAE, including studies [50,51] which showed that a depletion of TGFβ-activated kinase 1, an important signaling molecule for the activation of microglia, suppresses microglia from attaining an activated amoeboid morphology as well as reducing production of the pro-inflammatory cytokines IL-1β and chemokine (C-C motif) ligand 2 (CCL2). This cytokine suppression has been seen to result in a subdued EAE system with reduced immune cell infiltration and a lower level of demyelination [52]. The nuclear factor kappa- B (NF-κB) modulatory protein, A20, has also been identified as playing a regulatory role in microglial activation during EAE, with its deletion leading to hyperactivation of the NLRP3 inflammasome and causing increased levels of IL-1β [53]. 

Collectively, these studies suggest that microglial activation promotes and worsens the severity of EAE, and suppression of microglial activation appears to slow EAE disease progression entirely, leading to a less severe EAE clinical outcome. Furthermore, a recent 2018 study showed that inhibition of IL-1β and IL-18 generation by the caspase-1 inhibitor VX-765 blocks the activation of the NLRP3 inflammasome and prevents cytokine secretion, producing a less severe EAE disease outcome [45]. This is consistent with a self-perpetuating inflammasome response in chronic disease, which is disrupted with connexin hemichannel block [16].

Astrocytes also play major roles in the complex cellular cascade of events which occur throughout the CNS in response to injury or disease [54,55,56,57,58]. In both EAE and MS, astrocyte reactivity is a prevalent feature that begins during the early stages of the disease [46,59,60,61,62]. EAE astrocyte activation, as assessed by elevated GFAP immunoreactivity, cellular hypertrophy or gene expression, is recognized from the very early stages of disease onset, occurring in both grey and white matter [46,55,63]. Astrogliosis is a well-accepted measure of EAE progression and correlates with the severity of the EAE clinical outcome. Commencing prior to clinical symptomology, EAE astrocyte reactivity progressively intensifies, reaching a maximum during acute phases of the disease, mostly in the presence of immune cell infiltration, and persists throughout the chronic disease stages, then to a lesser extent [64,65]. We found that tonabersat significantly reduced astrocyte reactivity and was associated with a less severe clinical outcome during the chronic stages of our MOG_35–55_ EAE model.

This study has focused on changes to the innate immune system’s inflammasome pathway. Future work could delve into biochemical changes resulting from tonabersat treatment, and in particular, cerebral and serum levels of relevant inflammatory cytokines. Ongoing work also focuses on further exploring the morphological changes of glial cells in tonabersat-treated MOG_35–55_ EAE-affected animals and the human MS brain. Future work could also investigate the impact of tonabersat treatment on the adaptive immune system responses, such as T and B cell infiltration. Nonetheless, tonabersat has been taken through toxicity and carcinogenicity studies and has been used in Phase 2 clinical trials for the potential treatment of migraine [66]. With its connexin hemichannel block mode of action clarified and animal model clinical signs efficacy demonstrated in the present study, its use could be translated into clinical trials for the treatment of MS.

## 4. Materials and Methods

### 4.1. Animals

All experiments were approved and performed in accordance with the regulations of the University of Melbourne Animal Care and Use Standards Committee (ACUSC) (Approval number: 1714222.3) and the ARRIVE guidelines for animal research. Mice aged 6 weeks were moved from the Animal Resource Centre (Western Australia) to acclimate in the Biological Resource Facility (BRF) at the University of Melbourne prior to EAE induction. EAE was then induced in 8-week-old C57BL/6 male and female mice (equal number (*n* = 5) of both sexes in each group), and behavioral scoring was conducted over subsequent days, following which the animal brains were collected and embedded in paraffin and shipped to the University of Auckland for subsequent studies. The experimenter was blinded to avoid any potential bias during the behavioral and immunohistochemistry experiments, image acquisition, and analysis. Numbers 1–80 were allocated to each animal before the start of the experiment at the University of Melbourne by a person not involved in the experimental work. The mice and tissue sections were randomized following standard simple randomization procedures in a blinded fashion. The experimenter was unblinded when all data were collected and analyzed.

### 4.2. EAE Model Preparation

The detailed methods used for the EAE procedure are as described previously [25]. Briefly, EAE was induced by immunization of mice with 200 mg of MOG_35–55_-CFA solution (Myelin Oligodendrocyte Glycoprotein peptide in Complete Freund’s Adjuvant) injected subcutaneously near the inguinal lymph nodes and the axillary lymph nodes on the left side of the mouse, followed by an intraperitoneal injection administering 500 ng of Pertussis toxin (95008-6906; List Biological Laboratories Inc., Campbell, CA, USA) in phosphate-buffered saline (PBS), pH 7.4, on days 0, 2, and 7. Repeat intraperitoneal injections were always carried out on the opposite side of the previous injection. Mice were anaesthetized with a 2–2.5% isoflurane with an oxygen flow of 2 L per minute to minimise pain and distress. Tonabersat was administered via single daily intraperitoneal injections at doses of 0.2, 0.4 and 0.8 mg/kg, either following early dosing (days 3–15 post-inoculation) or late dosing (days 8–15 post-inoculation) regime. The mice were scored and checked daily for general health as per the Intervention Criteria Sheet and Monitoring. Clinical behavioral scoring was performed in accordance with the EAE clinical disease scoring system adapted from Pettinelli [67] and Hasselman [25] (Supplementary Table S1). Symptom onset typically occurred between days 7–15 (after the last set of MOG_35–55_ injections), with an increase in clinical scores between days 8–18. Behavioral tests were performed on all mouse groups and behavioral analysis followed clinical scoring guidelines; each mouse was given a clinical score corresponding to a set of behavioural traits daily. Normal naïve control (Control) mice consistently scored 0 throughout the experiment. Each experimental group’s daily average score was calculated for the 18 days following inoculation. During EAE procedures, several mice were culled (8 MOG_35–55_ and 7 tonabersat-treated mice) as the clinical score endpoint of 4.0 was reached or there was abdominal swelling. Twenty-eight mouse brains of the initial 80 underwent full immunohistochemical examination. This selection included groups A, B, and E (Supplementary Table S2).

### 4.3. Tissue Processing

Mice were euthanized using carbon dioxide inhalation and the brains were immediately removed using a fine pair of tweezers to remove the skull beginning at the cerebellum. The brains were then placed in 4% neutral buffered formalin overnight and subsequently transferred to the pathology laboratory for paraffin embedding.

The paraffin-embedded mouse brain blocks were left on ice to cool for 20 min, then loaded into a paraffin microtome (Leica RM2235, Heidelberg, Germany), and 7 μm coronal sections were slowly trimmed then collected using ice-cold forceps before being laid into a 37 °C water bath until they flattened. A single ribbon of three sections at a time was mounted onto a positively charged microscopy slide (Grade HDS). These steps were repeated for the whole block, with the cutting beginning from the olfactory bulb to the spinal cord. A total of 10–20 series of 10 slides, each containing three coronal sections, were collected and left to dry at room temperature (RT).

### 4.4. Immunohistochemistry

Fluorescent immunohistochemistry was used to examine the expression of MBP, NLRP3, Iba-1 and GFAP. Paraffin-embedded brain sections were placed on a heating block at 60 °C for 1 h to melt the paraffin wax before being cleared in xylene (2 × 5 min) and rehydrated in 100% ethanol twice for 5 min, followed by washes in Milli-Q H_2_O for 5 min. Heat-induced antigen retrieval was performed in sodium citrate buffer (10 mM sodium citrate, 0.05% tween 20, pH 6.0) in a pressure cooker (2100 Antigen Retriever, Aptum Biologics Ltd., Southampton, Hampshire, UK) for 2 h at 121 °C. The slides were washed in PBS for 3 × 5 min and permeabilised in PBS containing 0.1% triton x-100 (PBS-T) for 15 min at 4 °C. Once dried, the sections were outlined using a hydrophobic barrier pen, ensuring the confinement of any dispensed liquid to the tissue and incubated in 10% normal goat serum (NGS) at RT for 1 h to block for non-specific binding of the secondary antibodies. The serum was aspirated, and the sections were incubated in primary antibody (Supplementary Table S3) diluted in 1% NGS overnight in a humidified chamber at 4 °C. The following day, the slides were washed for 3 × 5 min in PBS before being incubated with Hoechst 33342 nuclear counterstain (1:10,000, H3570; Thermo Fisher, Waltham, MA, USA) and secondary antibodies (Supplementary Table S3) diluted (1:500) in 1% NGS at RT for 3 h. After a final 3 × 5 min of PBS washes, the slides were dried and cover-slipped with Mowiol (Calbiochem, San Diego, CA, USA) mounting medium, left to dry overnight at RT, and sealed with nail varnish. Negative control sections with the primary antibody omitted were run in tandem with each experiment. The omission of the primary antibodies resulted in a complete absence of immunoreactivity.

Serial sections from MOG_35–55_ EAE and control mice were stained for Iba-1 and GFAP to examine the level of neuroinflammation throughout the brain regions and determine the most affected areas. We found significant increases in microglia and astrocyte activation in all brain regions of MOG_35–55_ EAE mice, with the corpus callosum being the most affected. Therefore, our primary focus was to examine the expression of MBP, NLRP3, Caspase-1, Iba-1 and GFAP within the corpus callosum and in the two adjacent brain areas, the motor cortex and striatum within paraffin-embedded mouse brain sections of control, MOG_35–55_ EAE, and MOG_35–55_ EAE mice with 0.8 mg/kg tonabersat treatment. The ‘Mouse Brain in Stereotaxic Coordinates Second Edition Atlas’ was used to ensure accurate identification of the correct tissue plane. Plates corresponding to coordinates, Interaural 3.10 mm, Bregma −0.70 mm, were used for the identification of the corpus callosum, motor cortex and striatum.

### 4.5. Imaging and Analysis

The immunohistochemical experiments were performed on six sections per brain, and the two best-matching sections from the targeted planes were imaged. Images of the region of interest in both the right and left hemispheres of each section were captured and analysed. The final calculated result was an average of both the right and left-side measurements. Imaging was conducted on a Nikon Ni-E microscope equipped with a Nikon DS-Qi2 camera (Nikon, Tokyo, Japan). The rain regions were distinguished based on cell type and relative location with the use of Hoechst staining. Representative images were captured with a Nikon Plan Fluor 10×/0.30 objective, except NLRP3 images, which were captured with a Nikon Plan Fluor 20×/0.50 and Nikon Plan Fluor 40×/0.75 objective lens, respectively. The corpus callosum for the MBP staining was imaged at multiple regions of interest to cover the entire region. Three to four images spanning the entire corpus callosum region per hemisphere for each section were captured, which were first averaged within a hemisphere before being averaged between hemispheres for the final density value. ImageJ Fiji software version 1.47 (U.S. National Institutes of Health, Bethesda, MD, USA) was used to analyze the activated cell counts of Iba-1 and GFAP, and the staining/integrated densities of MBP, Iba-1, and GFAP in the corpus callosum, motor cortex, and striatum of the mouse brain. The final value was calculated as staining density or activated cell number divided by the area of the corpus callosum. For the motor cortex and striatum, density and activated cell number measurements were undertaken for a defined area of interest, measuring 2,979,718 μm^2^ in the motor cortex and 3,690,960 μm^2^ in the striatum. Activated glial cell counts were measured by selecting a particle size range with ImageJ Fiji 1.47 (U.S. National Institutes of Health, Bethesda, MD, USA). Cells with activated morphology tend to be larger, with a larger cell body and an increased number of primary processes [31,68,69,70]. Particles between 1 and 699 pixels were classified as small particles, and particles between 700 and 20,000 pixels were classified as large particles and recognized as activated cells for both Iba-1 (microglia) and GFAP (astrocytes). The accuracy of the measurement was optimized and validated by visual assessment of cell activation before conducting the automated measurement.

### 4.6. Statistical Analysis

Data in all experiments are expressed as mean ± SEM. All statistical analyses were conducted using GraphPad Prism version 9.0 (GraphPad Software Inc., San Diego, CA, USA). Multiple-measure two-way ANOVA was used to assess the behavioral scores, and one-way ANOVA followed by Tukey’s post-hoc testing was used to examine differences between the different groups in all other experiments with a *p*-value of *p* ≤ 0.05 considered significant. Adobe Photoshop CC 2017 (Adobe Systems Software, San Jose, CA, USA) was used to prepare the figures.

## 5. Conclusions

In summary, the findings of this study show that NLRP3 inflammasome activation and neuroinflammation were heavily implicated in our MOG_35–55_ EAE animals. Modulation of these processes with the Cx43 hemichannel blocker, tonabersat, significantly reduced inflammation and prevented or delayed demyelination by preventing NLRP3 inflammasome activation. This dramatically improved the clinical outcome of MOG_35–55_ EAE-affected animals. Previous studies and clinical work have highlighted the safety profile in humans of the clinical trial phase IIB-ready Cx43 hemichannel blocker, tonabersat, and it may merit further investigation as a safe pharmacological candidate for MS treatment. Tonabersat may significantly reduce disease progression and offer a disease-modifying treatment by reducing inflammation and brain lesions, slowing disability, and cutting relapses.

## Data Availability

All data generated or analysed during this study are included in this published article and its supplementary information files.

## Supplementary Materials

The following supporting information can be downloaded at: https://www.mdpi.com/article/10.3390/ijms242417454/s1.
