# Supplementary material for: Tonabersat Significantly Reduces Disease Progression in an Experimental Mouse Model of Multiple Sclerosis
Source: Int J Mol Sci. 2023 Dec 14;24(24):17454. doi: 10.3390/ijms242417454 (PMC10744318; doi:10.3390/ijms242417454)
Supplement: Supplementary file 1 [file ijms-24-17454-s001.zip › ijms-2775322-supplementary.pdf]

# Supplementary Materials

## Tonabersat Significantly Reduces Disease Progression in an Experimental Mouse Model of Multiple Sclerosis

**Andrea Kwakowsky [1,2,*], Bhavya Chawdhary [1,3,†], Antonio de Souza [1,3,†], Emily Meyer [1,3], Andrew H. Kaye [4,5], Colin R. Green [3], Stanley S. Stylli [4,6] and Helen Danesh-Meyer [3]**

[1] Department of Anatomy and Medical Imaging, Centre for Brain Research, Faculty of Medical and Health Science, University of Auckland, Auckland 1023, New Zealand; b.chawdhary@auckland.ac.nz (B.C.); antonio.desouza@auckland.ac.nz (A.d.S.); edan547@aucklanduni.ac.nz (E.M.)

[2] Pharmacology and Therapeutics, Galway Neuroscience Centre, School of Medicine, Ollscoil na Gaillimhe—University of Galway, H91 W5P7 Galway, Ireland

[3] Department of Opthalmology, Faculty of Medical and Health Sciences, University of Auckland, Auckland 1023, New Zealand; c.green@auckland.ac.nz (C.R.G.); h.daneshmeyer@auckland.ac.nz (H.D.-M.)

[4] Department of Surgery, University of Melbourne, Melbourne, VIC 3010, Australia; kaye.andrew@gmail.com (A.H.K.); stanley.stylli@mh.org.au (S.S.S.)

[5] Department of Neurosurgery, Hadassah Hebrew University Hospital, Jerusalem 91120, Israel

[6] Department of Neurosurgery, The Royal Melbourne Hospital, Parkville, VIC 3052, Australia

* Correspondence: andrea.kwakowsky@universityofgalway.ie; Tel.: +353-09149-3012

† These authors contributed equally to this work.

**Supplementary Figures**

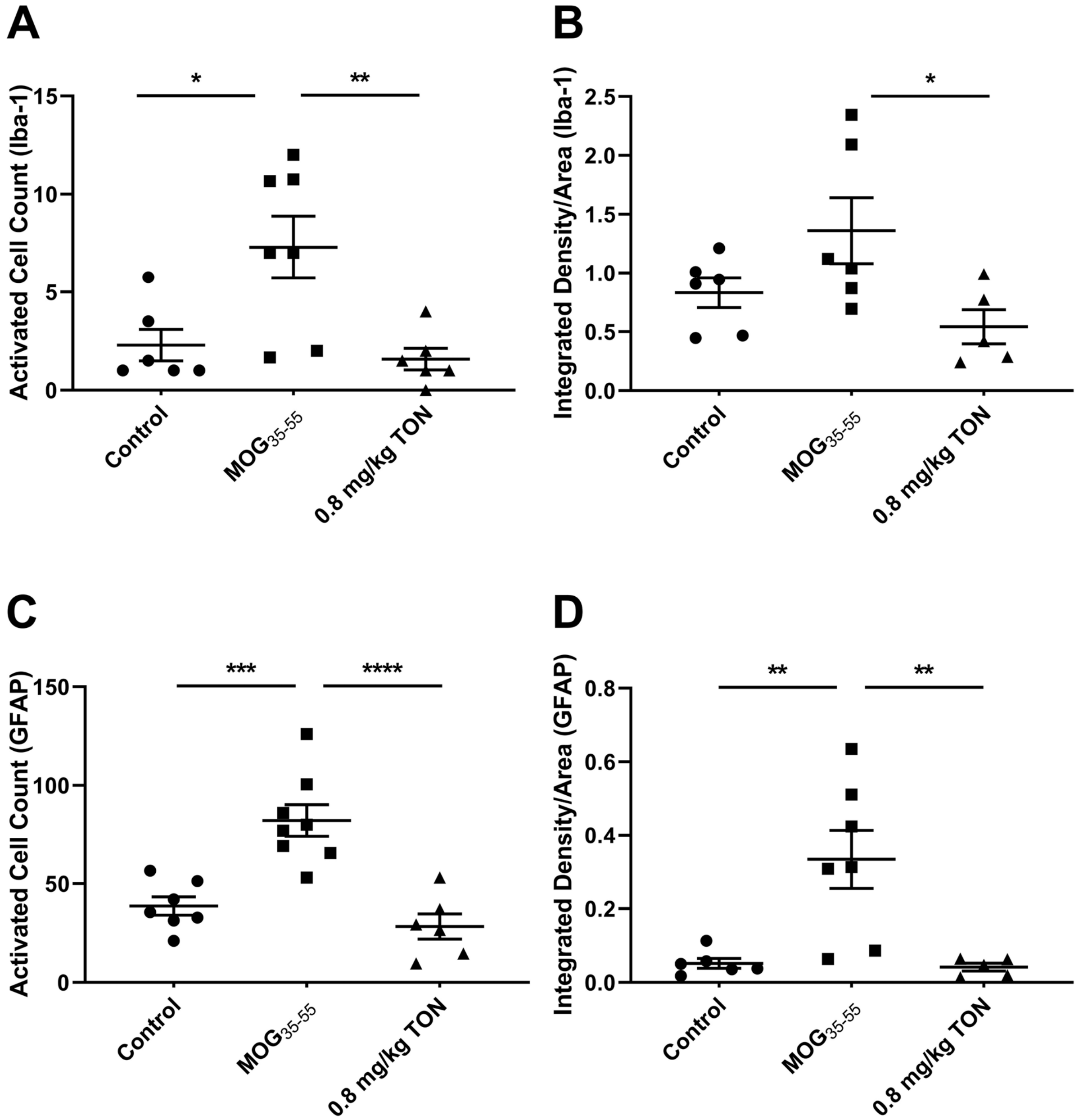


**Figure S1.** Mean activated cell count and mean integrated density of Iba-1 and GFAP in the motor cortex of control, $MOG_{35-55}$ EAE mice, and $MOG_{35-55}$ EAE mice with 0.8 mg/kg tonabersat treatment. The motor cortex of control mice, $MOG_{35-55}$ EAE ($MOG_{35-55}$) mice and $MOG_{35-55}$ EAE mice with 0.8 mg/kg tonabersat treatment (0.8 mg/kg TON) were stained with Iba-1 and GFAP. Activated microglia and astrocytic cell counts (**A**,**C**), and Iba-1 and GFAP integrated densities (**B**,**D**) of $MOG_{35-55}$ treated mice showed a significant increase compared to both control and 0.8 mg/kg TON mice. Control mice and 0.8 mg/kg TON mice shared similar means with no significant differences between the two groups for either inflammatory marker. Data points expressed as mean values ± SEM (one-way ANOVA and Tukey's post-hoc test (* $p \le 0.05$, ** $p \le 0.01$, *** $p \le 0.001$, **** $p \le 0.0001$; control mice $n$ = 6–7, $MOG_{35-55}$ treated mice $n$ = 7–8 and 0.8 mg/kg TON mice $n$ = 5–6)).

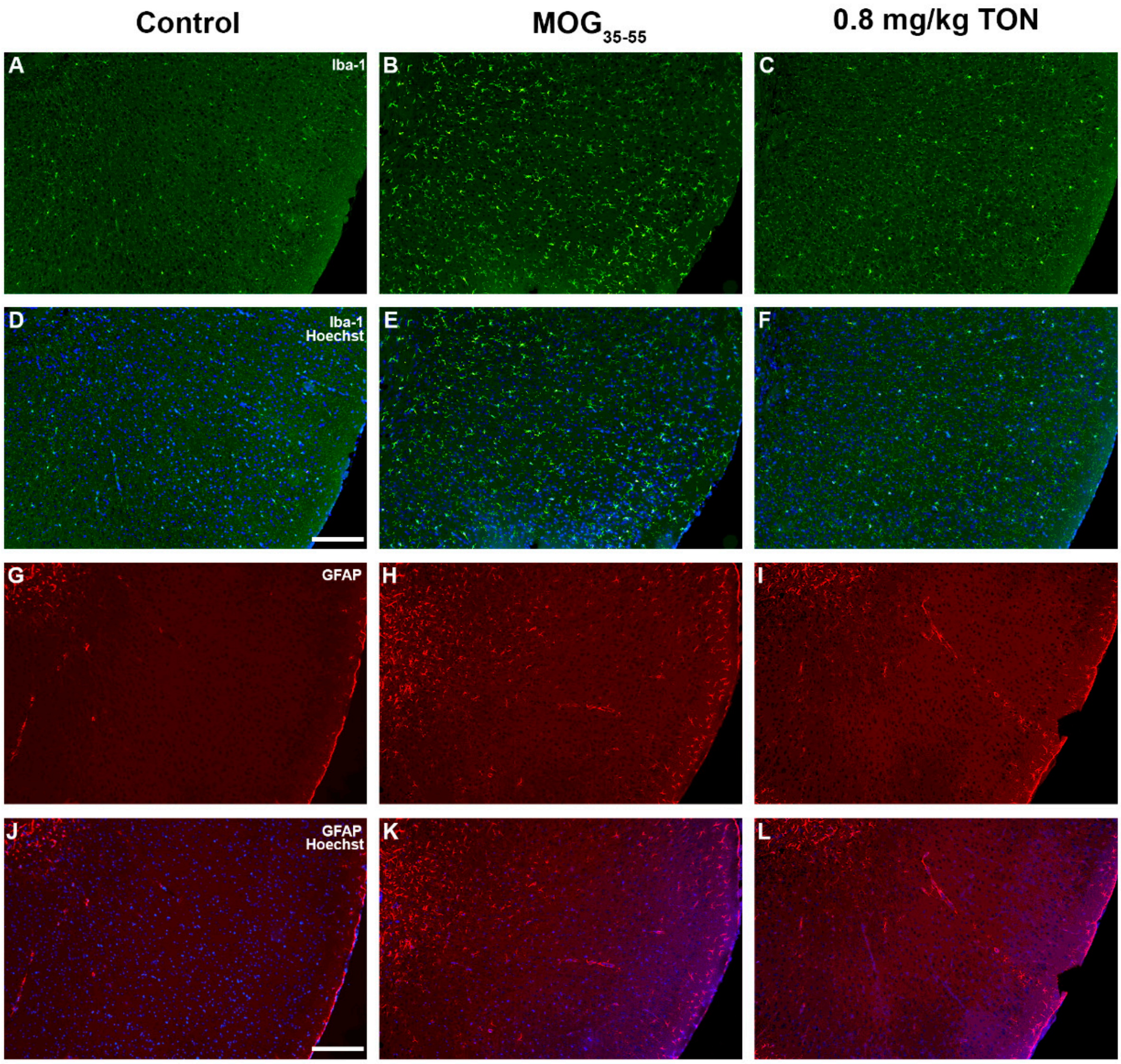


**Figure S2.** (**A**–**F**) Photomicrographs showing the effects of tonabersat on Iba-1 expression within the mouse motor cortex. Representative images depicting the immunolabeling of Iba-1 (green) and Hoechst (blue) within the motor cortex of the mouse brain in control (**A**,**D**), $MOG_{35–55}$ EAE ($MOG_{35–55}$) mice (**B**,**E**), and $MOG_{35–55}$ EAE mice with 0.8 mg/kg tonabersat treatment (0.8 mg/kg TON) (**C**,**F**). $MOG_{35–55}$ mice show the greatest level of Iba-1 expression, while the 0.8 mg/kg TON mice showed a significant reduction of inflammation that is parallel to control mice. Scale bar = 200 µm. (**G**–**L**) Photomicrographs showing the effects of tonabersat on GFAP expression within the mouse motor cortex. Representative images depicting the immunolabeling of GFAP (red) and Hoechst (blue) within the motor cortex of the mouse brain in control (**G**,**J**), $MOG_{35–55}$ mice (**H**,**K**), and 0.8 mg/kg TON mice (**I**,**L**). $MOG_{35–55}$ mice show the greatest level of GFAP expression, while the 0.8 mg/kg TON mice showed a significant reduction in inflammation analogous to control mice. Scale bar = 200 µm.

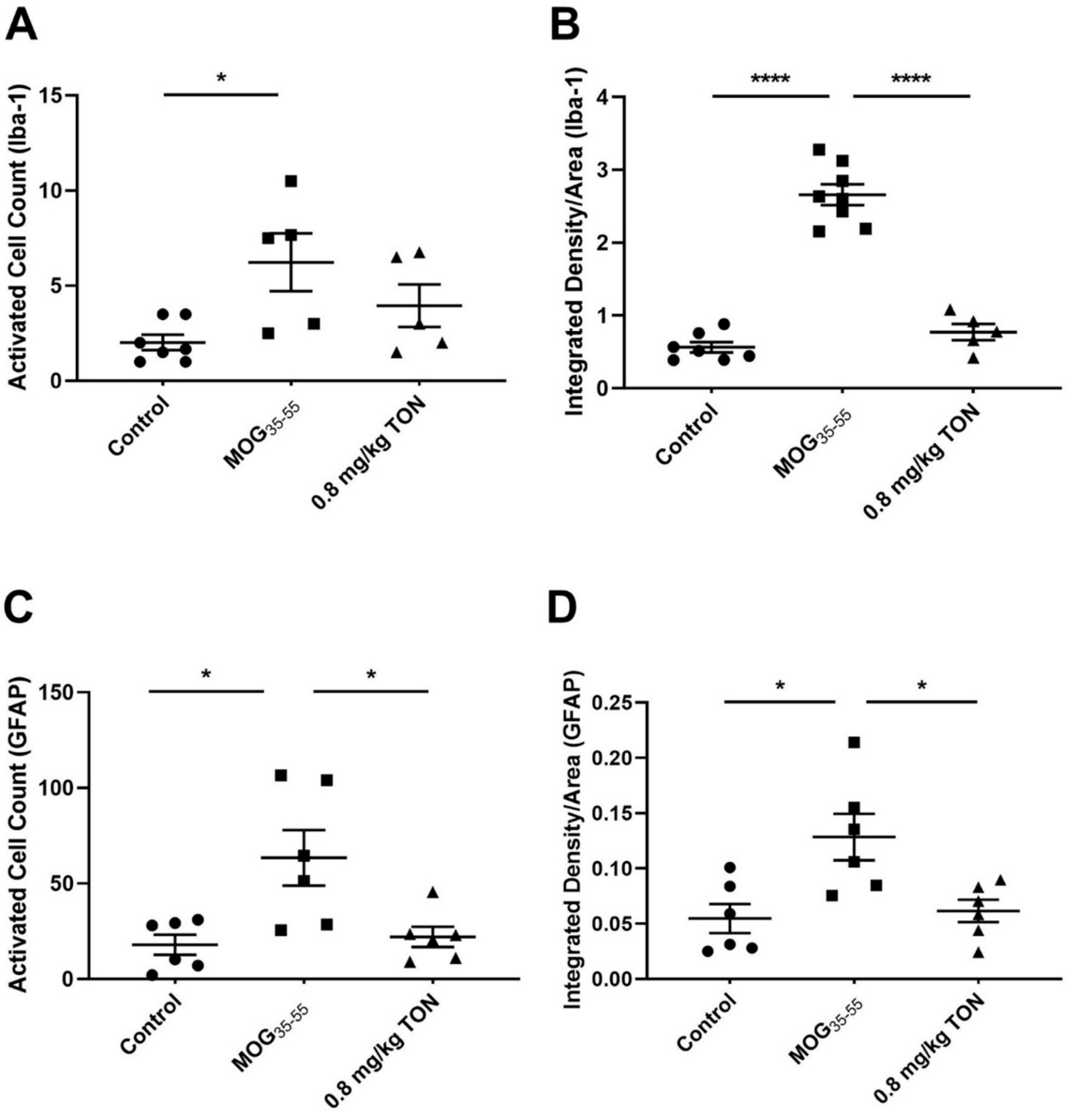


**Figure S3.** Mean activated cell count and mean integrated density of Iba-1 and GFAP in the striatum of control, $MOG_{35–55}$ EAE mice, and $MOG_{35–55}$ EAE mice with 0.8 mg/kg tonabersat treatment. The motor cortex of control mice, $MOG_{35–55}$ EAE ($MOG_{35–55}$) mice and $MOG_{35–55}$ EAE mice with 0.8 mg/kg tonabersat treatment (0.8 mg/kg TON) mice were stained with Iba-1 and GFAP. Activated microglia and astrocytic cell counts (**A**,**C**), and Iba-1 and GFAP integrated densities (**B**,**D**) of $MOG_{35–55}$ mice showed a significant increase compared to both control and 0.8 mg/kg TON mice. Control mice and 0.8 mg/kg TON mice shared similar means with no significant differences between the two groups for either inflammatory marker. Data points expressed as mean values ± SEM (one-way ANOVA and Tukey's post-hoc test (* $p \leq 0.05$, ****$p \leq 0.0001$; control mice $n$ = 6–7, $MOG_{35–55}$ treated mice $n$ = 7–8 and 0.8 mg/kg TON mice $n$ = 5–6)).

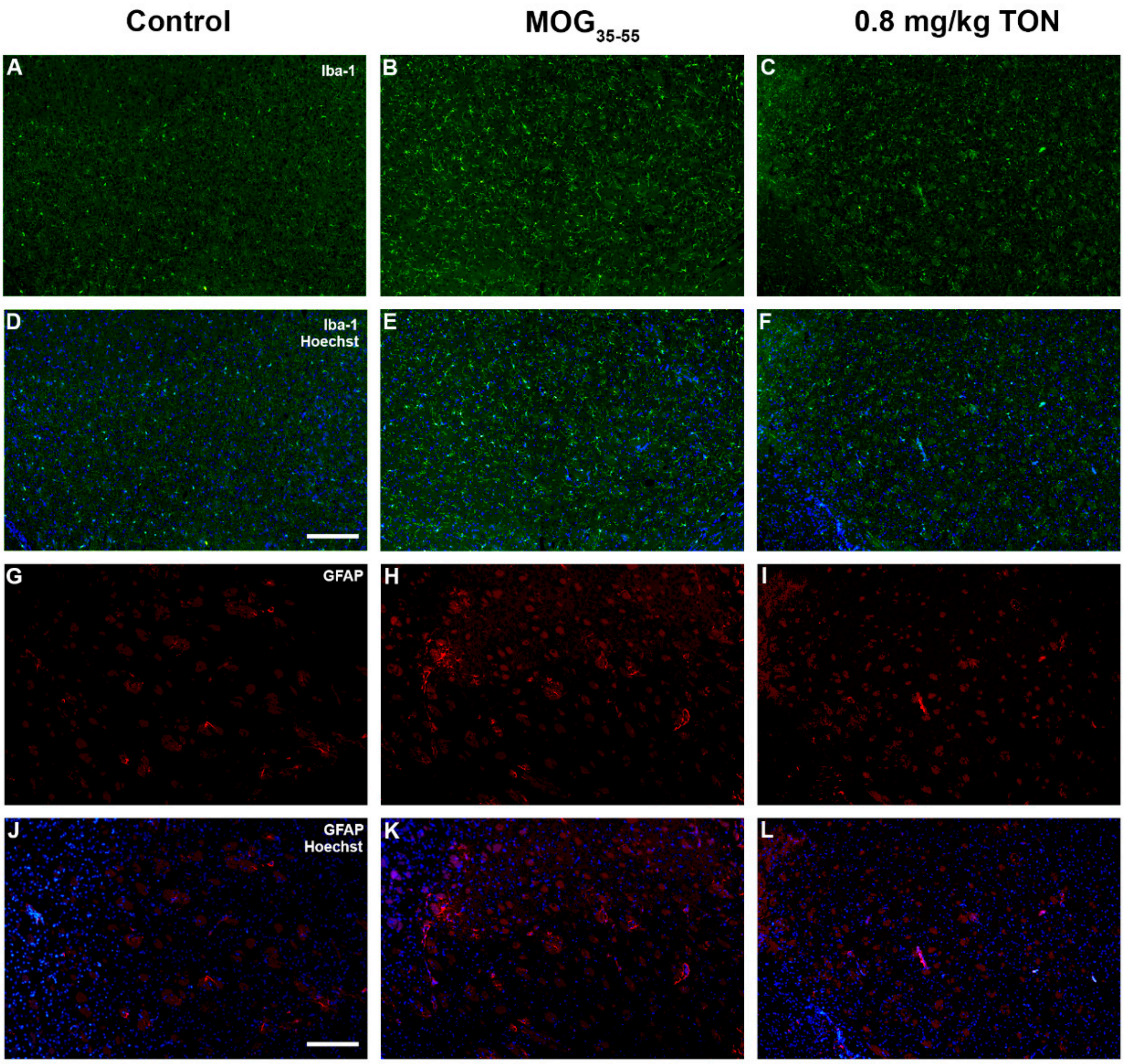


**Figure S4.** (**A**–**F**) Photomicrographs showing the effects of tonabersat on Iba-1 expression within the mouse striatum. Representative images depicting the immunolabeling of Iba-1 (green) and Hoechst (blue) within the striatum of the mouse brain in control (**A**,**D**), $MOG_{35–55}$ EAE ($MOG_{35–55}$) mice (**B**,**E**), and $MOG_{35–55}$ EAE mice with 0.8 mg/kg tonabersat treatment (0.8 mg/kg TON) (**C**,**F**). $MOG_{35–55}$ treated mice show the greatest level of Iba-1 expression, while the 0.8 mg/kg TON mice showed a significant reduction in inflammation that is comparable to control mice. Scale bar = 200 µm. (**G**–**L**) Photomicrographs showing the effects of tonabersat on GFAP expression within the mouse striatum. Representative images depicting the immunolabeling of GFAP (red) and Hoechst (blue) within the striatum of the mouse brain in control (**G**,**J**), $MOG_{35–55}$ mice (**H**,**K**), and 0.8 mg/kg TON mice (**I**,**L**). $MOG_{35–55}$ mice show the greatest level of GFAP expression, while the 0.8 mg/kg TON mice showed a significant reduction in inflammation that is analogous to control mice. Scale bar = 200 µm.

## Supplementary Tables

**Table S1.** Clinical EAE Score Sheet.

| Score | Scoring Method | Observation |
|---|---|---|
| 0 | Hold mouse by base of the tail | Unaffected; mouse can 'helicopter' tail |
| 0.5 | Hold mouse by base of the tail | Some loss of tail tone |
| 1.0 | Hold mouse by base of the tail | Complete tail limpness, with no evidence of limb weakness |
| 1.5 | Hold mouse at base of tail between index finger and thumb, resting the heel of your palm flat on a surface. Attempt to roll the mouse once by rolling its tail between your fingers. | Can roll mouse, but with some struggling |
| 2.0 | Hold mouse at base of tail between index finger and thumb, resting the heel of your palm flat on a surface. Attempt to roll the mouse once by rolling its tail between your fingers. | No hind limb paralysis upon ambulation, but mouse fails to remain upright when the examiner attempts to roll the mouse. |
| 2.5 | Hold mouse at base of tail and place its forepaws on edge of the cage. | Climbs into cage with difficulty; normal ambulation. |
| 3.0 | Hold mouse at base of tail and place its forepaws on edge of the cage. | Inability to climb over cage edge; partial paralysis of hind limbs; waddles upon ambulation (but does not drag limbs). |
| 3.5 | Observe ambulation | Partial paralysis of hind limbs as evidenced by dragging one limb upon ambulation. |
| 4.0 | Observe ambulation | Complete paralysis of both hind limbs; dragging body by forearms; still capable of moving around the cage. |

**Table S2.** Treatment groups in this study.

| Group | Treatment | Tonabersat Regime | Sample Size (*n*) |
|---|---|---|---|
| A | Control | N/A | 9 |
| B | $MOG_{35–55}$ | N/A | 9 |
| C | $MOG_{35–55}$ + 0.2 mg/kg TON | Early | 10 |
| D | $MOG_{35–55}$ + 0.4 mg/kg TON | Early | 10 |
| E | $MOG_{35–55}$ + 0.8 mg/kg TON | Early | 10 |
| F | $MOG_{35–55}$ + 0.2 mg/kg TON | Late | 10 |
| G | $MOG_{35–55}$ + 0.4 mg/kg TON | Late | 10 |
| H | $MOG_{35–55}$ + 0.8 mg/kg TON | Late | 10 |

**Table S3.** Primary antibodies used in the mouse immunohistochemistry experiments.

| Antigen | Immunogen | Company, Host Species, Catalogue no. | Dilution | Detection |
|---|---|---|---|---|
| Casp-1 (Cleaved Asp210) | A synthetic peptide derived from human Caspase 1 | Invitrogen, PA5-38099 | 1:100 | Goat anti-rabbit Alexa 488 (2110499) |
| GFAP | Glial Fibrillary Acidic Protein from pig spinal cord | Sigma-Aldrich, Mouse, C9205 | 1:5000 | Conjugated with Cy3 |
| Iba-1 | Synthetic peptide to recognize microglia | Abcam, Rabbit, Ab178846 | 1:20,000 | Goat anti-rabbit Alexa 488 (2110499) |
| NLRP3 | Synthetic peptide within human NLRP3 aa 1–100 conjugated to keyhole limpet haemocyanin | Abcam, Rabbit, Ab214185 | 1:100 | Goat anti-rabbit Alexa 647 (Thermo Fisher, A21245) |
| MBP | Cytoplasmic side of myelin | Novus Biologicals, Rabbit, NB600-717 | 1:4000 | Goat anti-rabbit Alexa 647 (Thermo Fisher, A21245) |

**Table S4.** Day 18 mean clinical scores in the $MOG_{35–55}$ EAE mice and each early and late tonabersat drug dosing groups.

| Dosing Group | Control Clinical Score | $MOG_{35–55}$ EAE Clinical Score | 0.2 mg/kg TON Clinical Score | 0.4 mg/kg TON Clinical Score | 0.8 mg/kg TON Clinical Score |
|---|---|---|---|---|---|
| Early Dosing | 0.0 | 3.7 ± 0.27 | 2.2 ± 0.15 | 1.8 ± 0.14 | 1.6 ± 0.10 |
| Late Dosing | | | 2.1 ± 0.16 | 1.8 ± 0.14 | 1.8 ± 0.15 |
